# Overexpression of BMP1 reflects poor prognosis in clear cell renal cell carcinoma

**DOI:** 10.1038/s41417-019-0107-9

**Published:** 2019-06-03

**Authors:** Wen Xiao, Xuegang Wang, Tao Wang, Jinchun Xing

**Affiliations:** 1 Department of Urology, The First Affiliated Hospital of Xiamen University, Xiamen, Fujian China; 2 Center of Diagnosis and Treatment of Urinary System Diseases, The First Affiliated Hospital of Xiamen University, Xiamen, Fujian China; 3 The Key Laboratory of Urinary Tract Tumors and Calculi of Xiamen City, The First Affiliated Hospital of Xiamen University, Xiamen, Fujian China; 4 The First Clinical College of Fujian Medical University, Xiamen, Fujian China

**Keywords:** Renal cancer, Biomarkers

## Abstract

Clear cell renal cell carcinoma (ccRCC) is the highest mortality, invasion, and metastasis subtype of renal cell carcinoma. Bone morphogenetic protein (BMP) family has recently emerged as a group of cancer-related proteins in multiple pathogenesis of cancers. Currently, little is known about the prediction role of BMPs in ccRCC. Therefore, we screened The Cancer Genome Atlas Kidney Clear Cell Carcinoma (TCGA-KIRC) database for ccRCC patients with complete clinical information and BMP family expression data. Multivariate analysis showed that high expression of BMP1 was associated with shorter overall survival (OS) (*P* = 0.001), and shorter disease-free survival (DFS) (*P* = 0.018). Gene set enrichment analysis (GSEA) showed BMP1 was associated with epithelial–mesenchymal transition (EMT), G2M checkpoint, angiogenesis, hypoxia pathway, and Kirsten rat sarcoma viral oncogene (KRAS) signaling. Knockdown BMP1 suppressed malignancy of ccRCC in vitro and in vivo. Our results indicated that high expressions of BMP1 were poor prognostic factors and gene therapy could be an effective treatment for ccRCC.

## Introduction

Renal cell carcinoma (RCC) accounts for 3.77% in adult malignancies, is one of the common lethal malignancy in urology with about 65,340 new cases and 14,970 deaths were estimated for 2018 in the United States [1] and with about 403,262 (2.2%) new cases and 175,098 (1.8%) deaths worldwide for all cancers in 2018 [2]. In the past two decades, the incidence of RCC has increased gradually and up to 30% patients have suffered from cancer metastasis when they were primarily diagnosed with RCC [3, 4]. Clear cell RCC (ccRCC) is still an extremely lethal disease and accounts for most cancer-related deaths [5]. Timely detection and early diagnosis is extremely important for the treatment management. Thus, it would be significant to illustrate the potential molecular mechanisms for genesis and development of ccRCC.

Management of different diagnosis and treatment of ccRCC is based on cancer progression. A total of 90% of patient deaths are associated with cancer metastasis and invasion [6, 7]. Tumor recurrence is still a high risk after nephrectomy for local-regional tumor patients [8]. Specific molecular target drugs based on the tyrosine kinase inhibitor (TKI) use benefits metastatic ccRCC patients [9, 10]. Unfortunately, ccRCC patients still develop TKI resistance and lead to poor clinical progression because of intrinsic resistance or acquired resistance of tumor cells [11]. Exploring effective prognostic molecular biomarkers and their molecular mechanisms in tumor progression and metastasis is critical to assessing and managing ccRCC patients.

Bone morphogenetic proteins (BMPs) were originally discovered as secreted cytokines based on their ability to induce bone. BMP was found to be an activator and member of transforming growth factor-beta (TGF-β) family. BMP1 originally identified as a secreted metalloprotease of the astacin metalloproteinase family [12]. TGF-β signaling pathway activation requires BMP1 activity, BMP1 makes the initial cleavage and release TGF-β complex from the matrix. The complex then was cleavage by other metalloproteinases such as MMP2 to free TGF-β. Hence, BMP1 involves in the activation of the TGF-β and BMP signaling pathways [13]. In recent years, BMP1 upregulation may increase cancer invasiveness in gastric cancer [14], lung cancers [15], osteosarcoma [16], and colon cancer [17]. Nevertheless, to the best of our knowledge, the involvement of BMP1 in ccRCC has not understood yet.

In this study, we investigated the expression of BMPs with clinicopathological features and patient survival in TCGA database, gene set enrichment analysis (GSEA) were used to explore the biological role which would be involved in ccRCC. In vitro and in vivo experiments certificated knockdown BMP1 suppressed malignancy of ccRCC. Our study demonstrated that BMP1 acted as an oncogene and high BMP1 expression predicts poor progression. These findings could serve as the foundation for developing new prognostic markers and eventually leading to better treatment efficacy.

## Patients and methods

### Patient samples

The Cancer Genome Atlas database of Kidney Clear Cell Carcinoma (TCGA-KIRC) contains total 533 patients. Eight patients were excluded as the clinical data were partly missing and 525 patients with complete clinical data were used for univariate and multivariate Cox proportional hazard regression [18]. Detailed clinical and molecular characteristics could be found on the TCGA website (http://cancergenome.nih.gov/). Overall survival (OS) and disease-free survival (DFS) were the primary end points of this study. OS was defined as the time between diagnosis and death or was censored at the last follow-up. DFS was defined as the time between diagnosis or surgery and the recurrence of disease or death (for any reason) at the last follow-up. Surgical specimens of ccRCC patients were obtained from the Department of Urology, the First Affiliated Hospital of Xiamen University between 2016 and 2018. Inform and obtain informed consent to approve the experimental and research procedures from the Xiamen University Institutional Review Board.

### RNA extraction and qRT-PCR

Tissue RNA was extracted with TRizol reagent (Thermo, MA, USA) according to the manufacturer’s instructions. The purity and concentration of the RNA was tested by a NanoDrop 2000 spectrophotometer (NanoDrop Technologies, Wilmington, USA). qPCR analysis was performed according to the manufacturer’s instructions (LightCycler 480II; Roche, Basel, Switzerland). Relative expression of BMP1 was calculated by: 2^−ΔCt^ (ΔCt = Ct_BMP1_–Ct_GAPDH_). Primers were purchased from GENEWIZ (GENEWIZ, Suzhou, China):

GAPDH (forward, 5′-GAGTCAACGGATTTGGTCGT-3′; reverse, 5′-GACAAGCTTCCCGTTCTCAG-3′)

BMP1, (forward, 5′-TGGCCGACTACACCTATGAC-3′; reverse, 5′- GGAGGACTTACGAGCTGTGT-3′)

### Oligonucleotide, lentivirus, plasmid, and shRNA

Oligonucleotides corresponding to the target sequences were annealed and cloned into the AgeI and EcoRI sites of the plko.l plasmid (Addgene, Cambridge, USA). Primers were listed as follows:

BMP1-1:

5′-CCGGCACCTCCCAGTACAACAACATCTCGAGATGTTGTTGTACTGGGAGGTGTTTTT-3′

5′-AATTAAAAACACCTCCCAGTACAACAACATCTCGAGATGTTGTTGTACTGGGAGGTG-3′

BMP1-2:

5′-CCGGGCGCTACTGTGGCTATGAGAACTCGAGTTCTCATAGCCACAGTAGCGCTTTTT-3′

5′-AATTAAAAAGCGCTACTGTGGCTATGAGAACTCGAGTTCTCATAGCCACAGTAGCGC-3′

### Cell culture and transient transfection

HK2, A498, Caki-1, and ACHN and came from The American Type Culture Collection (ATCC, USA) without recently authenticated. Cancer cells were cultured in high glucose DMEM medium (Wuhan Boster Biological Technology, Ltd, Wuhan, China) containing 10% FBS (Gibco; Thermo Fisher Scientific, Inc., Waltham, MA, USA) and antimycoplasma Plasmocin^™^ prophylactic (ant-mpp, InvivoGen, Hong Kong) at 37 °C, Under cultivation 5% CO_2_ incubator.

### Cell proliferation, migratory, and invasion assays

The A498 and Caki-1 cells were first transfected with sh-BMP-1, sh-BMP-2, or shRNA negative control plasmid (NC), and then the cells were added to the 96-well plate at a density of 3 × 10^3^/well. Cell proliferation rate (OD value) was determined by Cell Counting Kit-8 (CCK-8) (Dojindo Molecular Technologies, Inc., Rockville, MD, USA) according to the manufacturer’s instructions. Homogenized cells without plasma for 24 h, then 10^5^ cells were cultured in a 24-well transwell plate with polycarbonate membrane inserts (Corning, NY, USA) for the migratory assay. A total of 2 × 10^5^ cells were cultured in a 24-well transwell plate with Matrigel (Thermo Fisher Scientific, Waltham, USA) coated polycarbonate membrane inserts for the invasion assay. Incubation for 24 h, cells were fixed with 100% methanol, then stained with 0.05% crystal violet, and five random regions were counted and three independent experiments were conducted in a previous study [19].

### Western blotting

Tissues and cells are pyrolyzed in a protein lysis system containing RIPA, PMSF (Wuhan Boster Biological Technology, Ltd, Wuhan, China), and protease inhibitor cocktail (Roche Diagnostics, Indianapolis, IN, USA). Protein concentration was measured at 562 nm. A total of 30 μg of proteins were presented in SDS-PAGE gel system then separated and transferred to polyvinylidene fluoride (PVDF) membrane (EMD Millipore, Bedford, MA, USA) for 90 min. After transfer to the PVDF membrane, the membrane was blocked in PBS containing 5% skim milk for 1 h and then incubated with antibodies against BMP1 (1:1000; ab205394; Abcam Co., Ltd) and GAPDH (1:2000; BM3876; Wuhan Boster Biological Technology, Ltd, Wuhan, China) at 4 °C overnight. The next day the membranes were washed and incubated with secondary antibodies (1:5000; BA1020; Wuhan Boster Biological Technology, Ltd, Wuhan, China) at room temperature (25 °C) for 2 h. Finally, the membranes were washed and detected by Biosense SC8108 Gel Documentation System with GeneScope V1.73 software (Shanghai BioTech, Shanghai, China).

### Immunohistochemistry (IHC)

Five adjacent normal, ccRCC tissues, and six xenograft tumor were fixed in formalin, dehydrated, and embedded, and then the tissue sections were incubated with primary mouse BMP1 (1:100) and Ki67 (1:100; A11005; ABclonal Biotech Co., Ltd), overnight at 4 °C. The sections were washed three times with PBS, and then the sections were incubated with the secondary antibody for 2 h at room temperature.

### Xenograft tumor in nude mice

All animal experiments were performed in accordance with animal protocols approved by the Institutional Animal Use and Care Committee of Xiamen University. Six male mice (Beijing Hfk Bioscience, Beijing, China) were randomly divided into two groups. A total of 1 × 10^6^ Caki-1 cells with sh-BMP1-1 or NC were injected subcutaneously into the flanks of mice. The investigator was blinded to the group allocation during the experiment. Tumor size was measured every 3 days. After 30 days, the mice were euthanized and the tumors were harvested. Tumor volumes were calculated as follows: volume = (D × d^2^)/2.

### Bioinformatics analysis

GSEA is used to understand the BMP1 pathway involved in the pathogenesis of TCGA-KIRC [20]. Nominal *P* < 0.05 and a false discovery rate < 25% had considered to be significantly enriched for enriched gene sets analysis.

### Statistical analysis

Data sets were described by median and error bars as s.e.m. The RNA levels of samples were analyzed with unpaired-sample *t*-test by two-sided and *χ*^2^ tests with clinical data. The Kaplan–Meier (KM) curve evaluated survival rate for patients clinical and expression level of BMP1 with log-rank test. Prognostic significance of BMP1 was analyzed by univariate and multivariate Cox proportional hazard regression in ccRCC as previously described [21]. All objects are greater than or equal to three for statistical analysis. The variance is similar between the groups. *P* < 0.05 was considered to have statistical significance. All statistical analyses were performed by SPSS Statistics 22.0 (IBM SPSS, Chicago, IL).

## Results

### Relative expression of BMP family in ccRCC

**Fig. 1:**
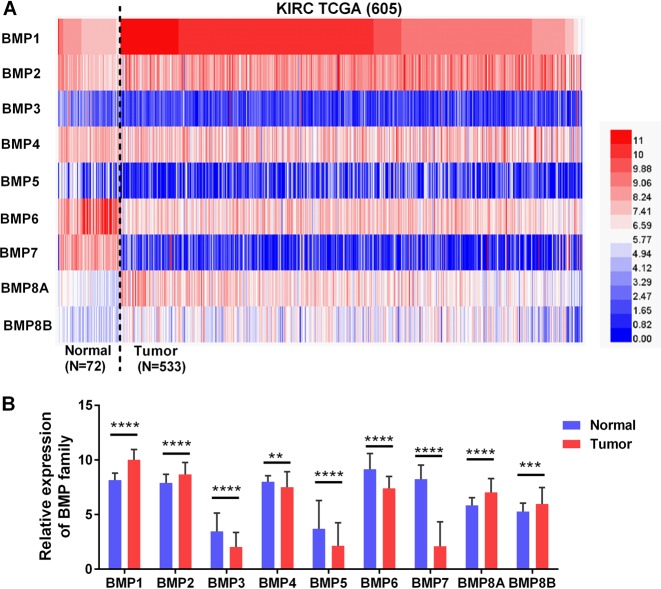
BMP family expression in TCGA-KIRC microarray datasets. **a** Heat map depicting BMPs expression in TCGA-KIRC microarray datasets (*n* = 605). **b** Relative BMPs expression in TCGA-KIRC. Red indicates high expression; white indicates medium expression; blue indicates low expression. BMP bone morphogenetic protein, TCGA-KIRC The Cancer Genome Atlas kidney renal clear cell carcinoma. **P < 0.01, ***P < 0.001, and ****P < 0.0001

To investigate the expression of BMP family in ccRCC development, we investigated the mRNA expression levels of the nine BMP family members (BMP 1–8 A and B) in TCGA database. The heat map showed the expression levels of BMP family members in Fig. 1a. The expression of each family members in ccRCC tissues and corresponding noncancerous normal tissues is shown in Fig. 1b. Among the nine BMP families, BMP1 exhibited the most obvious high expression in ccRCC (Fig. 1a, b).

### Prognostic significance of BMP family in ccRCC

**Fig. 2:**
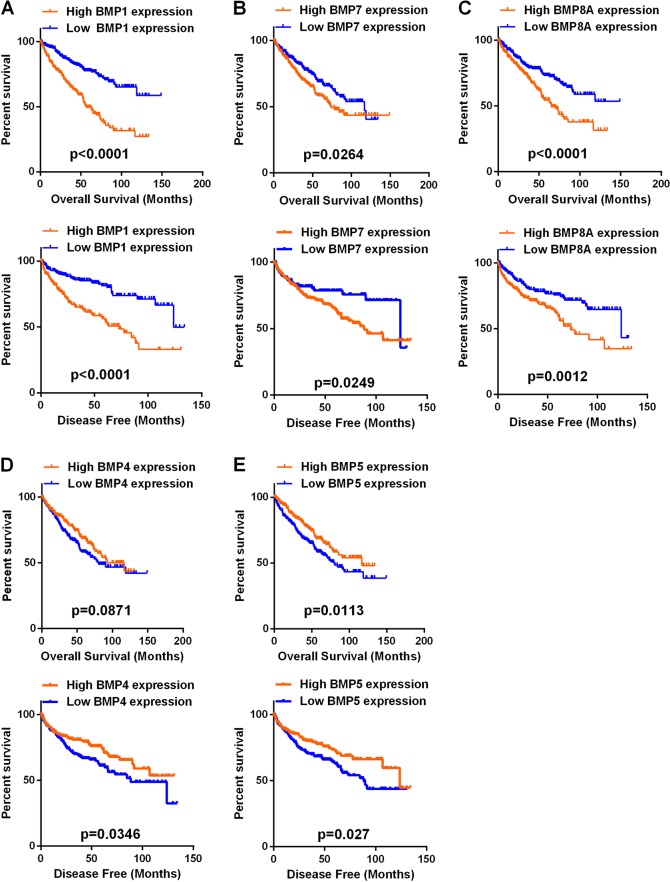
Kaplan–Meier curves of OS and DFS in different expression levels of BMP1, BMP4, BMP5, BMP7, or BMP8A. **a**–**c** High BMP1, BMP7, BMP8A expressers had shorter OS and DFS than the low expressers. **d** Low BMP4 expressers had shorter DFS than the low expressers. **e** Low BMP5 expressers had shorter OS and DFS than the low expressers. OS overall survival, DFS disease-free survival, BMP bone morphogenetic protein

According to the median expression levels of the nine BMP family members, all patients were divided into two groups. Univariate analysis showed that the expression of BMP1, BMP7, or BMP8A was associated with OS and BMP1, BMP4, BMP5, BMP7, or BMP8A was associated with DFS (Table 1). KM analysis demonstrated that the patients with high BMP1, BMP7, and BMP8A expression had shorter OS and DFS than those with low expression (Fig. 2a–c). High BMP4 expression group had longer DFS than the low expression group but no difference in OS (Fig. 2d). The high BMP5 expression group had longer OS and DFS than the low expression group (Fig. 2e). Then we compared the expression of each member in ccRCC tissues and corresponding noncancerous normal tissues, unfortunately we found that BMP4, BMP5, and BMP7 expression was lower in tumor tissues (Fig. 1a, b). Following these initial results, we then focused the statistical analysis on BMP1 and BMP8A to patient’s OS and DFS.

**Table 1:** Comparison of OS and DFS between different expression levels of BMP1–8B

	OS		DFS	
Variables	*χ*^2^	*P* value	*χ*^2^	*P* value
BMP1 (high versus low)	39.265	0.000	27.960	0.000
BMP2 (high versus low)	0.822	0.365	0.035	0.851
BMP3 (high versus low)	0.036	0.850	0.525	0.468
BMP4 (high versus low)	1.685	0.194	4.462	0.035
BMP5 (high versus low)	1.356	0.244	4.891	0.027
BMP6 (high versus low)	3.769	0.052	0.019	0.889
BMP7 (high versus low)	12.400	0.000	7.548	0.006
BMP8A (high versus low)	15.423	0.000	10.554	0.001
BMP8B (high versus low)	1.149	0.284	0.126	0.723

*OS* overall survival, *DFS* disease-free survival

### Clinical and molecular characteristics of the patients

Patients with integrity data (*n* = 525) were divided by BMP1 and BMP8A median expression levels respectively, detailed clinical pathologic information of these ccRCC patients was presented in Table 2. There was a significant association between high BMP1 expression and tumor stage (T stage), lymphatic metastasis (N stage), distant metastasis (M stage), TNM stages, and grade, but no significant differences in age or sex. Meanwhile, there was a significant association between high BMP8A expression and tumor stage (T stage), distant metastasis (M stage), and TNM stages, but no significant differences in age, sex, N stage, or grade.

**Table 2:** Correlation between BMP1 and BMP8A mRNA expression and clinicopathological parameters of ccRCC patients

		BMP1 mRNA expression				BMP8A mRNA expression			
Variables		Low (*n* = 263)	High (*n* = 262)	*χ*^2^	*P* value	Low (*n* = 263)	High (*n* = 262)	*χ*^2^	*P* value
Age (years)	<=60	122	138			126	134		
	>60	141	124	2.073	0.163	137	128	0.550	0.485
Sex	male	160	180			96	89		
	female	103	82	3.588	0.068	167	173	0.369	0.584
T stage	T1 + T2	194	143			192	143		
	T3 + T4	69	119	21.014	0.000	71	117	17.807	0.000
N stage	N0 + NX	260	250			257	253		
	N1	3	12	5.594	0.019	6	9	0.629	0.447
M stage	M0 + MX	240	207			235	212		
	M1	23	55	15.563	0.000	28	50	7.387	0.007
Grade	G1 + G2	147	98			126	119		
	G3 + G4	116	164	18.027	0.000	137	143	0.327	0.600
TNM stage	I + II	186	133			184	79		
	III + IV	77	129	21.930	0.000	135	127	18.709	0.000

### Multivariate analyses of OS and DFS

To assess the prognostic significance of the aforementioned clinical and molecular characteristics in ccRCC patients, we chose the expression levels of BMP1 and BMP8A (high versus low), age, sex, T stage, N stage, M stage, and grade to construct multivariate analyses of OS and DFS (Table 3 and Table 4). Multivariate analysis demonstrated that age (HR, 1.713; *P* = 0.001), T stage (HR, 1.6495; *P* = 0.034), N stage (HR, 2.166; *P* = 0.020), M stage (HR, 2.522; *P* = 0.000), Grade (HR, 1.607; *P* = 0.013), and BMP1 expression (HR, 1.903; *P* = 0.001) could be considered independent prognostic indicators of OS. Meanwhile, multivariate analysis demonstrated that T stage (HR, 1.856; *P* = 0.005), N stage (HR, 3.097; *P* = 0.002), M stage (HR, 5.050; *P* = 0.000), Grade (HR, 2.172; *P* = 0.000), and BMP1 expression (HR, 1.692; *P* = 0.018) could be considered independent prognostic indicators of DFS. Multivariate survival analyses indicated that the BMP1 expression was an independent prognostic factor for OS and DFS in ccRCC patients.

**Table 3:** Univariate and multivariate analyses of BMP1 and BMP8A mRNA level and patient overall survival

	Univariate analysis			Multivariate analysis^c^		
Variable	HR^a^	95% CI^b^	*P*	HR	95% CI	*P*
Overall survival						
Age (years)						
≤60 versus >60	1.803	1.318–2.468	0.000	1.713	1.252–2.344	0.001
Sex						
Female versus male	0.948	0.697–1.290	0.825			
T stage						
T3 or T4 versus T1 or T2	3.120	2.306–4.220	0.000	1.495	1.032–2.166	0.034
N stage						
N1 versus N0 or NX	3.832	2.070–7.061	0.000	2.166	1.132–4.415	0.020
M stage						
M1 versus M0 or MX	4.346	3.192–5.918	0.000	2.522	1.763–3.610	0.000
Grade						
G3 or G4 versus G1 or G2	2.639	1.885–3.697	0.000	1.602	1.106–2.321	0.013
BMP1						
High versus low	2.696	1.839–3.951	0.000	1.903	1.324–2.734	0.001
BMP8A						
High versus low	1.821	1.262–2.626	0.001	1.194	0.842–1.693	0.321

^a^Hazard ratio, estimated from Cox proportional hazard regression model

^b^Confidence interval of the estimated HR

^c^Multivariate models were adjusted for T, N, M classification, age, and gender

**Table 4:** Univariate and multivariate analyses of BMP1 and BMP8A mRNA level and patient disease-free survival

	Univariate analysis			Multivariate analysis^c^		
Variable	HR^a^	95% CI^b^	*P*	HR	95% CI	*P*
Disease-free survival						
Age (years)						
≤60 versus >60	1.366	0.959–1.945	0.084			
Gender						
Female versus male	1.413	0.951–2.100	0.087			
T stage						
T3 or T4 versus T1 or T2	4.526	3.134–6.538	0.000	1.865	1.209–2.877	0.005
N stage						
N1 versus N0 or NX	5.942	2.983–11.836	0.000	3.097	1.490–6.435	0.002
M stage						
M1 versus M0 or MX	8.529	5.877–12.379	0.000	5.050	3.316–7.691	0.000
Grade						
G3 or G4 versus G1 or G2	3.376	2.236–5.098	0.000	2.172	1.407–3.352	0.000
BMP1						
High versus low	2.696	1.839–3.951	0.000	1.692	1.097–2.622	0.018
BMP8A						
High versus low	1.820	1.262–2.626	0.001	1.240	0.823–1.871	0.304

^a^Hazard ratio, estimated from Cox proportional hazard regression model

^b^Confidence interval of the estimated HR

^c^Multivariate models were adjusted for T, N, M classification, age, and gender

### Biological pathogenesis of BMP1 in ccRCC

**Fig. 3:**
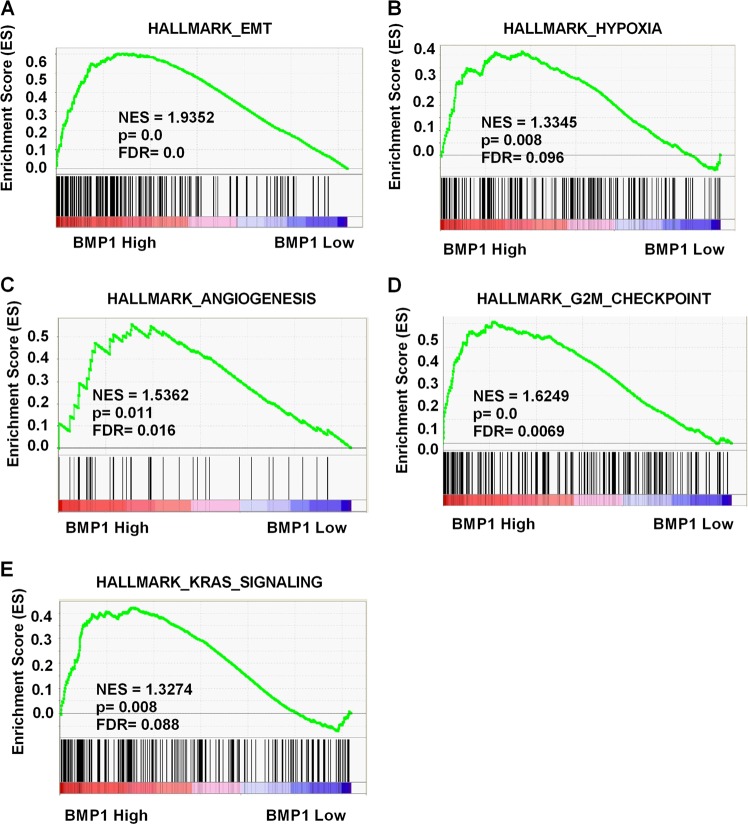
Pathway involved in the pathogenesis of BMP1 in TCGA-KIRC with GSEA. Enrichment curves are shown for activated gene sets related to **a** EMT, **b** hypoxia pathway, **c** angiogenesis, **d** G2M checkpoint, and **e** KRAS signaling. BMP bone morphogenetic protein, TCGA-KIRC The Cancer Genome Atlas kidney renal clear cell carcinoma, GSEA gene set enrichment analysis, EMT epithelial–mesenchymal transition, KRAS Kirsten rat sarcoma viral oncogene

As BMP1 was upregulated and an independent prognostic factor for OS and DFS in TCGA-KIRC database, we were eager to know how BMP1 was involved in ccRCC pathogenesis. Then we used GSEA, a computational tool to obtain statistically significant of the biological pathway in a database to a gene set. The results performed that the expression of BMP1 expression was associated with the gene signatures of epithelial–mesenchymal transition (EMT), hypoxia pathway, angiogenesis, G2M checkpoint, and Kirsten rat sarcoma viral oncogene (KRAS) signaling (Fig. 3).

### Downregulation of BMP1 suppresses malignancy of ccRCC in vitro

**Fig. 4:**
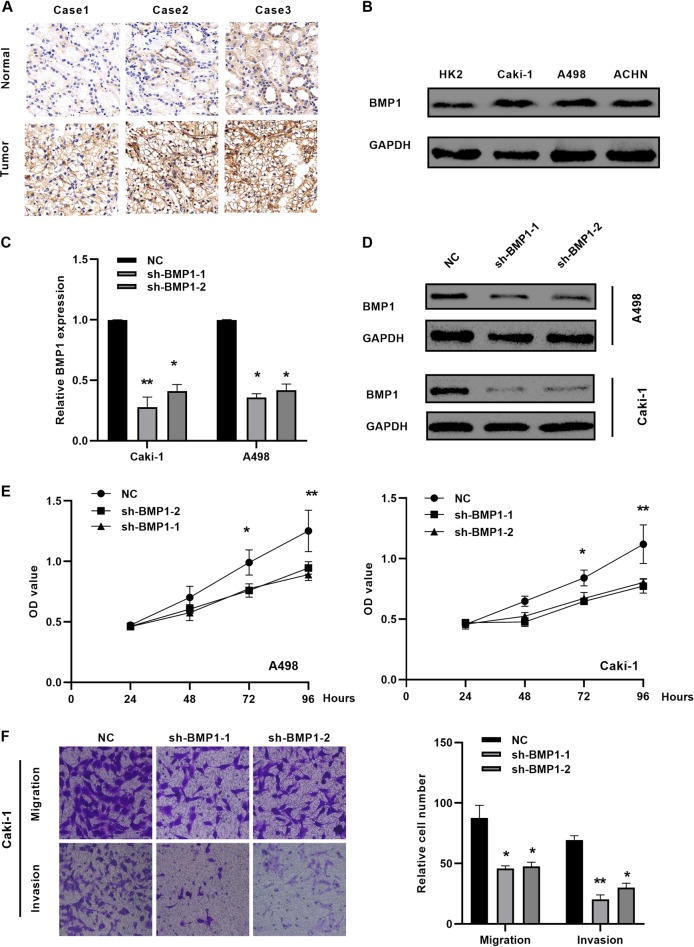
Downregulation of BMP1 suppresses malignancy of ccRCC in vitro. **a, b** BMP1 is upregulated in ccRCC tissues and renal cancer cells by immunohistochemistry. **c, d** Silence BMP1 with shRNA (sh-BMP1-1 and sh-BMP1-2). **e** Silence BMP1 significantly repressed the cell proliferation of A498 and Caki-1 cells. **f** Silence BMP1 significantly repressed the migration and invasion of Caki-1 cells. Data indicate the means ± SEM. *P < 0.05. **P < 0.01

As BMP1 mRNA was upregulated in TCGA-KIRC database, we confirmed the protein level in ccRCC cancer tissues and cells with western blotting (Fig. 4a, b), two BMP1 shRNA plasmids were transfected into the A498 and Caki-1 cells, resulting in a consistent BMP1 knockdown (Fig. 4c, d), CCK-8 and transwell experimental experiments revealed that BMP1 knockdown inhibited the proliferation, cell migration, and invasion in A498 and Caki-1 cells (Fig. 4e, f).

### The knockdown of BMP1 levels inhibits tumorigenicity in vivo

**Fig. 5:**
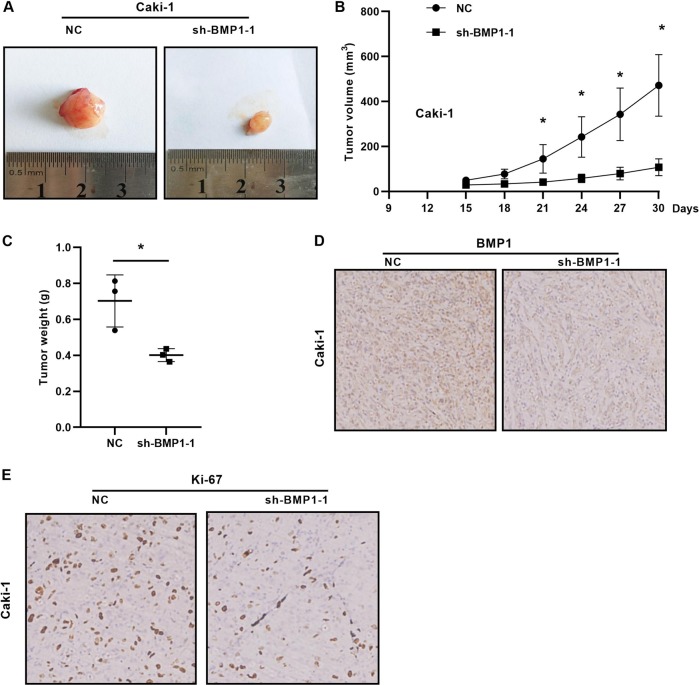
The knockdown of BMP1 levels inhibits tumorigenicity in vivo. **a**–**c** Tumor size, weight, and volume curves in the xenograft formation assay of BMP1 knockdown by Caki-1 cells. **d, e** The expression of BMP1 and Ki67 protein in xenograft tumor tissues. Data indicate the means ± SEM. *P < 0.05

We examined the effect of knockdown BMP1 in renal tumor growth in vivo. Caki-1 cells stably infected with lentiviral shRNA-BMP1 and implanted into flank of node mice. The tumor volume was measured every 3 days with the last measurement on day 30. We found that BMP1 knockdown in Caki-1 cells significantly reduced tumor volume and tumor weight (Fig. 5a–c). The expression of BMP1 and Ki67 protein in tumor tissues was shown in Fig. 5d, e, IHC staining indicated that knockdown BMP1 decreased Ki67 protein expression. Taken together, these results demonstrated that BMP1 reducing could inhibit renal cancer growth.

## Discussion

CcRCC accounted for ~80% of all RCC histological subtypes and had the highest mortality, invasiveness, metastatic rate, resistance to traditional radiotherapy and chemotherapy. This study reported the expression of nine BMP family members in the clinical significance of ccRCC and found that high expressions of BMP1 and BMP8A had prognostic effects on ccRCC, especially the BMP1 and biological functions for the first time.

Wang et al. [22] had shown that BMP2 inhibits tumor-initiating ability in human renal cancer stem cells. But we observed that BMP2 was upregulated in TCGA-KIRC database. This might be explained by the focus on all RCC of the previous study, while our study had specific focus on clear cell subtypes. Markic et al. [23] had shown that all BMPs (BMP2, BMP4, BMP6, BMP7) and their receptors (BMPRIA, BMPRIB, and BMPRII) mRNA have stronger expression levels in RCC, especially BMP2 is elevated strongly in kidney cancer. Lee et al. reported that BMP6 activated interleukin-10-mediated M2 polarization of tumor-associated macrophages to promote RCC growing. BMP6 was a marker signature associated with a poor prognosis in human RCC specimens [24]. BMP1 originally identified as a secreted metalloprotease but not a ligand of the BMP signaling pathway [12]. BMP1 makes the initial cleavage and releases TGF-β complex from the matrix, and then to free TGF-β. Hence, BMP1 involves in the activation of the TGF-β and BMP signaling pathways [13]. In recent years, BMP1 was found to be upregulated in gastric cancer [14], lung cancers [15], osteosarcoma [16], and colon cancer [17]. Nevertheless, the involvement of BMP1 in ccRCC has not been studied yet.

In this study, we investigated the expression of BMPs with clinicopathological features and patient survival in TCGA database, We found that: (I) BMP1 exhibited the most obvious high expression in ccRCC; (II) high BMP1 expression was significantly associated with T stage, N stage, M stage, TNM stages, and grade; (III) the BMP1 expression level was an independent predictor of prognostic of OS and DFS in TCGA-KIRC; (IV) high BMP1 expression was associated with the gene set of EMT, hypoxia pathway, angiogenesis, G2M checkpoint, and KRAS signaling with GSEA; and (V) downregulation of BMP1 suppresses malignancy of ccRCC in vitro and in vivo.

The normal function of von Hippel–Lindau (VHL) tumor suppressor can abolish the hypoxia-inducible transcription factor (HIF) and HIF acts as an important oncogene to promote renal tumor growth [25–28]. BMP1 and HIF1A were positively correlated with the malignant grade of astrocytomas [29], but no research reported their direct or indirect relationship. GSEA demonstrated that high BMP1 expression was associated with the hypoxia signaling pathways in patients with ccRCC, it may be hypothesized that BMP1 could positively regulated by hypoxia signaling, or even upregulated HIF expression in ccRCC, further experiments need to be verified.

Cancer progression by conferring a more motility and aggressive phenotype on cancer cells is a key foundation for EMT [30, 31]. Previous high-throughput screening has identified BMP1 RNA sequences as the most upregulated transcripts in human tumor endothelium associated with angiogenesis [32]. BMP1 was reported to activate TGF-β signaling by cleavage of latent TGF β -binding protein [33]. GSEA demonstrated that high BMP1 expression was associated with the gene signatures of EMT and angiogenesis in TCGA-KIRC database, it may be hypothesized that BMP1 could positively regulate EMT and angiogenesis in ccRCC. GSEA also demonstrated that higher BMP1 expression was associated with the gene signatures of G2M checkpoint and KRAS signaling, it may reveal that BMP1 could positively control cell cycle progression in ccRCC. We proved that knockdown BMP1 suppressed malignancy of ccRCC in vitro and in vivo.

This study is the first study to demonstrate the functional role of BMP1 in ccRCC tumor progression. These results also demonstrate that BMP1 can be used as a potential novel biomarker for predicting the prognosis of ccRCC patients. However, whether BMP1 can promote HIF expression or regulated by HIF does not investigated in ccRCC cells.

In conclusion, we investigated the role of BMP1 in ccRCC progression including TCGA database with univariate and multivariate Cox proportional hazard regression and GSEA. Our studies indicated that BMP1 expression is significantly upregulated and correlated with various clinicopathological parameters. High BMP1 expression is significantly positively correlated with disease progression, which identifies BMP1 as an independent predictor of prognosis in ccRCC patients and is involved in the development of renal cancer. All of these studies provided clues to understanding the potential role of BMP1 in ccRCC and suggested that downregulation of BMP1 may provide a new therapeutic strategy for the management and manipulation of ccRCC patients.
